# The Degrees of Coronary Heart Disease and the Degrees of New-Onset Blepharitis: A Nationwide Cohort Study

**DOI:** 10.3390/diagnostics14131349

**Published:** 2024-06-25

**Authors:** Chia-Yi Lee, Shun-Fa Yang, Yu-Ling Chang, Jing-Yang Huang, Chao-Kai Chang

**Affiliations:** 1 Institute of Medicine, Chung Shan Medical University, Taichung 40201, Taiwan; 2 Nobel Eye Institute, Taipei 100008, Taiwan; 3 Department of Ophthalmology, Jen-Ai Hospital Dali Branch, Taichung 41265, Taiwan; 4 Department of Medical Research, Chung Shan Medical University Hospital, Taichung 40201, Taiwan; 5 Department of Medical Education, Cathay General Hospital, Taipei 106438, Taiwan; 6 Department of Optometry, Da-Yeh University, Chunghua 51591, Taiwan

**Keywords:** coronary heart disease, blepharitis, age, severity, epidemiology

## Abstract

In this study, we aimed to evaluate the association between the severity of coronary heart disease (CHD) and the subsequent severity of blepharitis. This retrospective population-based cohort study was conducted using the National Health Insurance Research Database (NHIRD) of Taiwan. The participants with a CHD diagnosis were divided into mild CHD and severe CHD groups at a 1:2 ratio, according to whether percutaneous coronary intervention (PCI) was performed. The main outcomes were the development of blepharitis and severe blepharitis with the application of antibiotics. Cox proportional hazard regression was performed to obtain the adjusted hazard ratio (aHR) for blepharitis, with a 95% confidence interval (CI) between the groups. There were 22,161 and 15,369 blepharitis events plus 9597 and 4500 severe blepharitis episodes in the mild and severe CHD groups, respectively. The severe CHD group showed a significantly higher incidence of blepharitis development (aHR, 1.275; 95% CI: 1.051–1.912, *p* = 0.0285), whereas the incidence of severe blepharitis was not significantly different between the groups (aHR, 0.981; 95% CI: 0.945–1.020, *p* = 0.3453). The cumulative probability of blepharitis was significantly higher in the severe CHD group than in the mild CHD group (*p* < 0.001). In the subgroup analyses, the correlation between severe CHD and blepharitis was more significant in patients older than 70 years compared to the younger group (*p* = 0.0115). In conclusion, severe CHD is associated with a higher incidence of blepharitis than mild CHD, and this correlation is more prominent in individuals older than 70 years.

## 1. Introduction

Coronary heart disease (CHD) is a vascular disorder attributed to coronary arterial stenosis and myocardial ischemia [1]. Individuals with a mild CHD type often present with a chronic course that can be treated by medicines such as anti-platelet agents and anti-lipid agents [2,3]. Severe CHD, however, can cause prominent coronary arterial occlusion that requires surgical management in order to prevent mortality [4]. Percutaneous coronary intervention (PCI) can be used to extend the narrowed arterial cavity in patients with severe CHD; therefore, the popularity of PCI has increased in recent years [5,6]. 

In addition to the heart and coronary arteries, CHD is also associated with certain disorders [7,8,9]. Behcet’s disease, a systemic inflammatory disease, can increase the incidence of CHD and other coronary artery impairments [10,11]. In addition, a previous study revealed an association between periodontitis and the incidence and severity of CHD [12]. In addition, there is a strong association between CHD and metabolic syndromes such as hypertension and diabetes mellitus (DM) [13,14]. Regarding the relationship between ophthalmic diseases and CHD, CHD has been associated with a higher risk of open-angle glaucoma [15].

Blepharitis is a common eyelid disease whose features include the chronic inflammation of the eyelid and meibomian gland dysfunction (MGD) [16]. Regarding co-morbidity, the existence of blepharitis is correlated with a higher incidence of metabolic syndromes, such as DM and dyslipidemia [17]. Few studies have evaluated the correlation between CHD and blepharitis. In addition, the association between the severity of CHD and severe blepharitis with the application of antibiotics remains unknown. Because CHD and blepharitis share a similar pathophysiology, with both exhibiting features of dyslipidemia and inflammation [1,16], it is possible that there is a relationship between these two diseases; this suggestion warrants further investigation.

Consequently, the purpose of the present study was to investigate the possible correlation between the severity of CHD and the subsequent severity of blepharitis. Our statistical analysis included several risk factors that are commonly associated with blepharitis.

## 2. Materials and Methods

### 2.1. Data Source

In this study, we used the National Health Insurance Research Database (NHIRD) of Taiwan as the data source; this database contains the medical records of approximately 23 million Taiwanese people from 1 January 2000 to 31 December 2020. The applicable records in the NHIRD were as follows: the International Classification of Diseases Ninth Revision (ICD-9) diagnostic code, the International Classification of Diseases Tenth Revision (ICD-10) diagnostic code, age, sex, occupation, income level, education level, urbanization of locality, image codes, laboratory codes, medical department codes, procedure codes, surgical codes, and international ATC codes for medicines paid by the health insurance system in Taiwan.

### 2.2. Participant Selection

**Figure 1:**
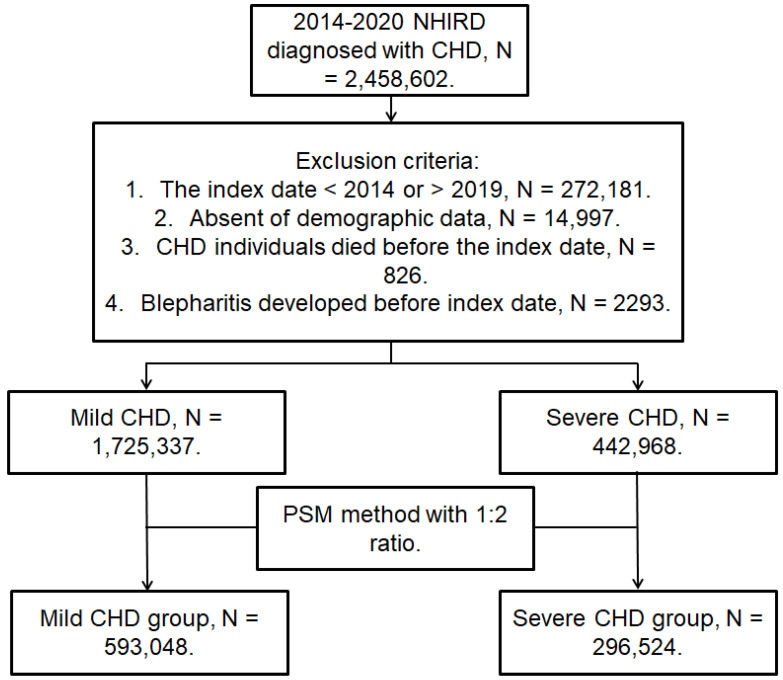
The flowchart of participant selection. CHD, coronary heart disease; N, number; NHIRD, National Health Insurance Research Database; PSM, propensity score matching.

This was a retrospective cohort study. Participants were defined as having CHD if they met the following inclusion criteria: (1) the receipt of CHD diagnoses according to ICD-9/ICD-10 codes from 2014 to 2019; (2) the arrangement of complete blood cell count, white blood cell differentiation count, cholesterol, triglyceride, high-density lipoprotein and low-density lipoprotein count, electrocardiogram, and cardiac angiography before the CHD diagnosis; (3) age from 20 to 100 years; and (4) visit and follow-up at the internal medicine, family medicine, or cardiovascular departments for more than three months. The index date of the present study was six months after CHD diagnosis. On the other hand, the following exclusion criteria were adopted: (1) an absence of demographic data; (2) the death of a CHD patient before the index date; (3) an index date later than 2019 or earlier than 2014; and (4) the occurrence of blepharitis before the index date. To evaluate the influence of the CHD severity, individuals with CHD were divided into mild CHD participants who received medications and those with severe CHD who received PCI management. One participant with severe CHD who underwent PCI was matched to another two participants with mild CHD received medications using the propensity score-matching (PSM) method. The PSM considered the demographic data, systemic disorders, and co-medications administered to patients with severe CHD and those with mild CHD. After PSM, 296,524 and 593,048 individuals were enrolled in the severe and mild CHD groups, respectively. A flowchart of the participant selection process is shown in Figure 1.

### 2.3. Main Outcome

The main outcomes of our study were the development of blepharitis and severe blepharitis that required treatment with antibiotics. Patients were considered to have blepharitis if they (1) received blepharitis-related ICD-9 and ICD-10 diagnostic codes; (2) underwent slit-lamp biomicroscope examination before the blepharitis diagnosis according to the exam code; and (3) were diagnosed with blepharitis by an ophthalmologist. Severe blepharitis was defined as (1) meeting the criteria for general blepharitis and (2) the prescription of topical or oral antibiotics according to the ATC codes. Only blepharitis events that emerged after the index date were considered as main outcomes. The participants in the present study were monitored until they developed blepharitis, were withdrawn from the National Health Insurance project or when the time limit of the NHIRD was met (31 December 2020).

### 2.4. Confounding Factors

Certain demographics, systemic diseases, and medications were included in the multivariable model in order to adjust for the effect of possible confounders on the development of blepharitis; these included age, sex, occupation, DM, hypertension, hyperlipidemia, cerebrovascular disease, peripheral vascular disease, rheumatoid arthritis, Sjogren’s syndrome, non-steroid anti-inflammatory drugs, systemic corticosteroids, biguanides, dipeptidyl peptidase-4 inhibitors, glucagon-like peptide-1 agonists, sodium-glucose cotransporter-2 inhibitors, and statins. The definitions of these confounding factors were in accordance with the demographic codes, ICD-9 and ICD-10 diagnostic codes, and ATC codes in the Taiwan NHIRD. To confirm that the periods of the confounding factors used in the present study were capable of changing the incidence of blepharitis, only confounding factors that had been documented for more than one year before the index date were included in the present study.

### 2.5. Statistical Analysis

SAS version 9.4 (SAS Institute Inc., Cary, NC, USA) was used for the statistical analyses in the present study. First, descriptive analyses were used to compare the demographics, systemic comorbidities, and medications administered in the mild and severe CHD groups, and the absolute standardized difference (ASD) was applied to evaluate the distribution of basic characteristics between the two groups. An ASD value higher than 0.1 was regarded as a significant difference. Subsequently, Cox proportional hazard regression was used to present the adjusted hazard ratios (aHR) and the 95% confidence intervals (CIs) for blepharitis and severe blepharitis episodes in the severe and mild CHD groups. The possible influence of the demographic data, systemic comorbidities, and medications was adjusted in the Cox proportional hazard regression. Kaplan–Meier curves were used to demonstrate the cumulative probability of blepharitis and severe blepharitis occurring between the two groups, and the log-rank test was used to compare the cumulative probability between the two groups. In the subgroup analyses, the participants with CHD were classified according to their age and sex; furthermore, Cox proportional hazard regression was administered again to investigate the aHR and 95% CI of blepharitis and severe blepharitis in individuals with severe CHD compared to patients with mild CHD in different subgroups. Subsequently, an interaction test was employed to illustrate the different correlations between the severity of CHD and the severity of blepharitis in each age- and sex-based subgroup. The statistical significance was determined to be *p* < 0.05, and a *p* value of less than 0.001 was depicted as *p* < 0.001 in the present study.

## 3. Results

The clinical characteristics of the mild and severe CHD groups are presented in Table 1. The sex ratio was identical between groups, as 65.05% of the participants in both groups were men (ASD = 0.0000), and the distribution of age was also similar between the mild and severe CHD groups (ASD = 0.0009). There were no significant differences between the two groups regarding individuals’ occupation and systemic comorbidities (all ASD < 0.1), and there was an insignificant difference in the number of medications prescribed between the mild and severe CHD groups due to the PSM procedure (all ASD < 0.1) (Table 1).

**Figure 2:**
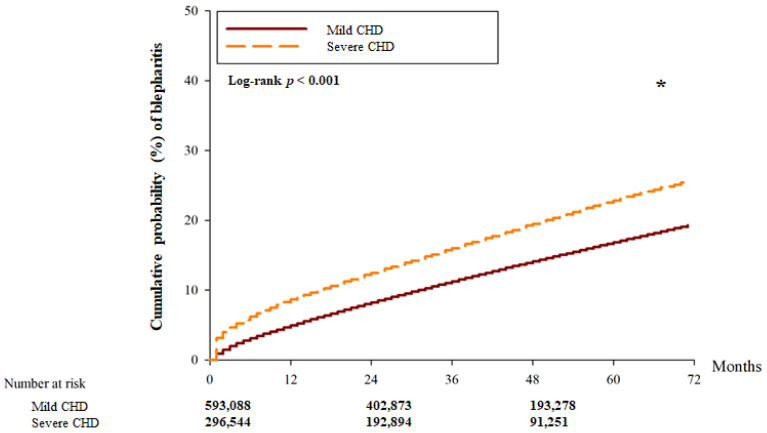
The cumulative incidence of blepharitis between the two groups. CHD: coronary heart disease. * denotes significant differences between the two groups.

**Figure 3:**
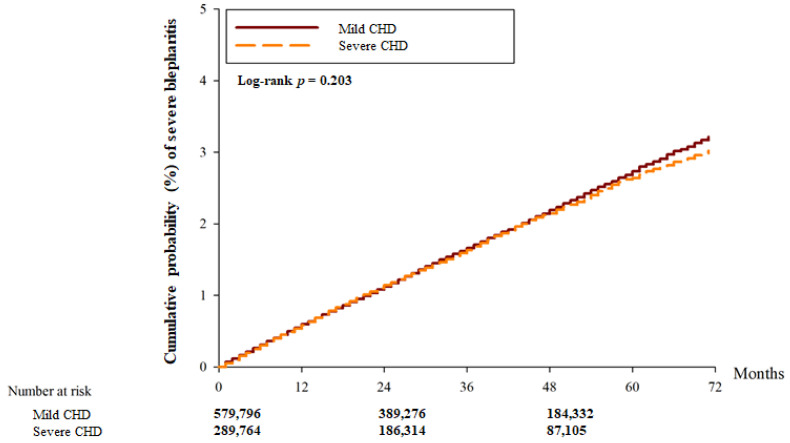
The cumulative incidence of severe blepharitis between the two groups. CHD: coronary heart disease.

There were 22,161 and 15,369 blepharitis events in the mild CHD and severe CHD groups, respectively. Another 9597 and 4500 severe episodes of blepharitis were observed in the mild CHD and severe CHD groups, respectively (Table 2). After adjusting for all possible confounding factors in the Cox proportional hazard regression, the severe CHD group showed a significantly higher incidence of blepharitis (aHR: 1.275; 95% CI: 1.051–1.912, *p* = 0.0285) than the mild CHD group. However, there was no significant difference in the incidence of severe blepharitis between the two groups (aHR: 0.981; 95% CI: 0.945–1.020, *p* = 0.3453). In addition, the cumulative probability of blepharitis was significantly higher in the severe CHD group than in the mild CHD group (*p* < 0.001) (Figure 2), whereas the cumulative incidence of severe blepharitis was similar between the two groups (*p* = 0.203) (Figure 3).

In the subgroup analyses, females with severe CHD showed a significantly higher incidence of blepharitis than females with mild CHD (aHR: 1.303; 95% CI: 1.064–2.043); meanwhile, the incidences of blepharitis in men with severe CHD and mild CHD were similar (aHR: 1.160, 95% CI: 0.960–1.592). The correlations between severe CHD and severe blepharitis were insignificant in both the male and female populations (*p* = 0.4571) (Table 3). Regarding the age-based subgroup analysis, patients with severe CHD older than 70 years presented a significantly higher incidence of blepharitis than mild CHD patients older than 70 years (aHR: 1.432, 95% CI: 1.201–2.171), and the correlation between severe CHD and blepharitis was more significant in patients aged >70 years than in those aged <70 years (*p* = 0.0115). The differences in the incidence of severe blepharitis between severe and mild CHD were insignificant in all age subgroups (all 95% CIs included 1) (Table 4).

## 4. Discussion

In brief, the present study demonstrated the significant correlation between severe CHD and the subsequent development of blepharitis in comparison to patients with mild CHD. In addition, it was found that this correlation is more prominent in patients older than 70 years. Moreover, the cumulative incidence of blepharitis was significantly higher in the severe CHD group than in the mild CHD group. On the other hand, the association between the severity of CHD and the subsequent development of severe blepharitis was insignificant.

The development of CHD is induced by several mechanisms and has been associated with an elevated risk of several co-morbidities in previous publications [8,18,19]. The pathophysiology of CHD comprises an adipose-tissue-related reaction and a high level of serum lipids; thus, obesity is also significantly associated with the development of CHD [20,21]. Another pathophysiology of CHD is the hyperglycemic status associated with insulin resistance and the development of CHD [22]. The presence of adipose tissue leads to the release of cytokines, such as interleukin and adiponectin, which contribute to low-grade chronic inflammation and the formation of atherosclerosis during the development of CHD [20]. The inflammatory response is one major pathway that contributes to the development of CHD; in this state, the level of cytokines, such as interleukin and C-reactive protein in the plasma, is significantly higher in individuals diagnosed with CHD [23], and the plasma matrix metalloproteinase-9 is an inflammatory marker that can serve as a predictor of CHD development [24]. In addition, the neutrophil-to-lymphocyte ratio is an inflammatory marker that correlates to the development of CHD and can serve as a predictor [25]. CHD is also related to the development of certain systemic inflammatory diseases, such as inflammatory bowel disease and ankylosing spondylitis [26,27]. On the other hand, dyslipidemia is another pathophysiology of CHD that exhibits a significant correlation with the formation of an atherosclerotic plaque and the subsequent development of coronary artery stenosis [28]. A higher level of LDL is a well-known risk factor for the development of CHD [29], and atherosclerotic plaque and the subsequent development of CHD could result from a higher level of triglyceride [29]. In addition, a high level of LDL is correlated with a higher risk of developing CHD, even in patients younger than 55 years old [21]. In addition to dyslipidemia and inflammation, there is a significant increase in oxidative stress in individuals with CHD, and the elevated oxidative stress represents a predictive factor for acute cardiovascular morbidities [30]. 

On the other hand, blepharitis is an eyelid disorder that is associated with the pathophysiologies of bacterial infection and inflammation [31], and chronic low-grade inflammation caused by bacterial components has often been observed in individuals with blepharitis [31]. The abnormality of lipids in the eyelid is one of the main pathophysiologies of blepharitis, and the presence of Propionibacterium acne is frequently associated with the development of blepharitis [32]. Chronic blepharitis may be associated with other inflammatory ophthalmic co-morbidities, such as conjunctivitis, chalazia, and hordeolum [33,34]. Regarding the relevant inflammatory markers, the elevated expression of cytokines at the ocular surface, such as interleukin-17, has been reported in individuals with Demodex blepharitis [35]; in addition, pro-matrix metalloproteinase-9 and interleukin are significantly increased in the tear film of individuals with blepharitis [36]. In addition to the inflammatory response, blepharitis is associated with the dysfunction of the meibomian gland and impaired lipid secretion [16]; furthermore, the presence of meibomian gland dysfunction and chronic blepharitis are significantly correlated with increased serum levels of LDL [37]. In addition, the presence of blepharitis is significantly associated with the subsequent development of dyslipidemia [17]; however, the application of statin could reduce the risk of developing blepharitis [38]. Thus, CHD and blepharitis have a similar pathophysiology that mainly includes inflammation and dyslipidemia [18,31], with some biomarkers such as LDL, interleukin, and matrix metalloproteinase increasing in the presence of CHD and blepharitis [21,24,36]. Moreover, diet modifications, such as supplementation with omega-3 fatty acids, could benefit the cardiovascular system and eyelid [39,40]; in addition, cardiovascular disease correlates with a higher risk of dermatitis and rosacea, which could contribute to the development of blepharoconjunctivitis [16,41,42]. Furthermore, severe CHD could reduce mobility and make daily clearance more difficult; this could result in poor lid and skin hygiene (a risk factor for blepharitis). Consequently, we speculate that the severity of CHD is associated with the subsequent development of blepharitis of different severities. This hypothesis is partially supported by the results of the present study.

In the present study, severe CHD is associated with a higher incidence of blepharitis. In a previous study, blepharitis was reported to be common in patients with hyperlipidemia and ischemic heart disease [34]. Nevertheless, the time sequences associated with CHD and blepharitis have not been made clear in previous research, and the correlation between the severity of CHD and the development of blepharitis has not been evaluated [34]. To our knowledge, this may be the first study to demonstrate that there is a potential correlation between the severity of CHD and the subsequent development of blepharitis. The major outcome we used in this study was the occurrence of blepharitis 6 months after the diagnosis of CHD; thus, the time sequence between CHD diagnosis and the subsequent development of blepharitis could be established. Moreover, several potential risk factors of blepharitis included DM and hyperlipidemia, and the medications prescribed for DM and hyperlipidemia were adjusted in the multivariable analysis [34,43]. Consequently, the severity of CHD may be an independent risk factor for the subsequent development of blepharitis. In addition, the cumulative probability of blepharitis was significantly higher in patients with severe CHD than those with mild CHD. This may indicate that the correlation between the severity of CHD and the development of blepharitis increases relative to the duration of severe CHD. On the other hand, severe CHD did not result in a higher probability of developing severe blepharitis, and the cumulative incidence of severe blepharitis between the severe CHD group and mild CHD group was also similar. One possible explanation for this phenomenon is that severe blepharitis is usually associated with the involvement of microorganisms that require treatment with antibiotics, and low periocular hygiene is also a prominent risk factor for severe blepharitis [16,31]. Consequently, the severity of CHD may have little influence in such a condition.

Regarding the subgroup analyses, females with severe CHD showed a significantly higher incidence of blepharitis compared to females with mild CHD. On the other hand, males with CHD did not exhibit a significantly higher incidence of blepharitis than those with mild CHD. In a previous study, sex did not represent a factor predisposing individuals to the development of blepharitis [34]. One possible explanation for this is that females can exhibit a higher risk of developing severe CHD compared to their male counterparts [44]. Since we only used the arrangement of PCI as the index for severe CHD, the female population in the present study may have exhibited a higher severity of CHD than the male population, even though both of them received PCI in order to manage the disease. Accordingly, the association between the severity of CHD severity and the development of blepharitis may be more prominent in the female population due to their CHD exhibiting greater severity. Nonetheless, the correlation between the severity of CHD severity and the development of blepharitis in the different sex subgroups was extremely similar according to the results of the interaction test. In addition, sex may not lead to a significant alteration in the correlation between the severity of CHD and blepharitis. For age-based subgroup analyses, the patients with severe CHD and older than 70 years old exhibited a higher incidence of blepharitis, with a significant aHR and a 95% CI higher than the mild CHD patients in the same age group. Furthermore, the correlation between the severity of CHD and blepharitis in this age subgroup was significantly stronger than that in the younger populations based on the interaction test. Because old age is a risk factor for blepharitis [45], the significant association between an older age and blepharitis in patients with severe CHD is reasonable. There was an insignificant correlation between severe CHD and severe blepharitis in all the subgroup analyses, which corresponds to the results of the whole-group analyses.

Concerning the epidemiology of this disease, CHD is a frequent disorder that has been found to affect nearly 5 percent of the white population in previous studies [46,47]. In eastern Asia, the prevalence of CHD is estimated to be higher than 25 percent [48]. Although this prevalence has decreased, severe CHD with advanced coronary atherosclerosis continues to develop in nearly 30 percent of all individuals with CHD [49]. The presence of CHD led to a considerable mortality rate of more than 40 percent in a population-based study conducted in the European population [50]. On the other hand, blepharitis is also a prevalent disease that affects approximately 38.9 percent of the general population [16]. Despite the fact that blepharitis rarely causes legal blindness, the presence of blepharitis is associated with the development of MGD, dry eye disease, and corneal lesion in previous study [16]. In previous publications, dry eye disease has been found to affect approximately 75 percent of people older than 40 years and lead to a significant reduction in individuals’ quality of life [51]. Moreover, dry eye disease is an underestimated pathology that can cause visual impairment and distress [51,52]. In addition, the corneal ulcers, superficial keratopathy, and corneal neovascularization caused by blepharitis can lead to visual loss [53]. Thus, both CHD and blepharitis affect a significant number of people and could contribute to major economic costs. It is therefore important to diagnose blepharitis in a timely manner in order to avoid subsequent ocular damage in specific populations, such as those with CHD; thus, the results of the present study may be demonstrated.

The present study has several limitations. Firstly, we used the data obtained from databases rather than real clinical documents to perform the analyses conducted in the present study. Accordingly, many pieces of important data, including details regarding the examination, clinical presentation, treatment, and prognosis of CHD and blepharitis, are not available in the present study. In addition, the retrospective design of the present study diminishes the homogeneity of the study population, despite the application of the PSM method. In addition, the arrangement of PCI for CHD and the application of antibiotics for severe blepharitis depend on the experience of the physician and their standard; thus, using these two forms of management as the severity indexes for CHD and blepharitis may not offer total accuracy. Some patients may have presented with diffuse long stenosis, which is an advanced form of CHD, but not have been indicated for PCI intervention in Taiwan; thus, the categorization method used in the present study may have wrongly categorized these patients into the mild CHD group rather than the severe CHD group. Finally, we excluded a considerable number of CHD cases during the exclusion process, which may have affected the statistical analyses. Nevertheless, the reason for the exclusion of such cases was the absence of critical examinations in most cases, and the elevation of the diagnostic accuracy may have compensated for the loss of participants.

## 5. Conclusions

In conclusion, the presence of severe CHD is correlated with the subsequent development of blepharitis than those with mild CHD, especially in patients older than 70 years. Furthermore, the development of blepharitis is associated with the duration of severe CHD. Consequently, periodical ocular examination may be recommended to those with severe CHD so that the underlying presence of blepharitis may be found. Further large-scale prospective studies are needed in order to evaluate the correlation between the severity of CHD and the prognosis of blepharitis after treatment.

## Figures and Tables

**Table 1:** The clinical characteristics of the mild and severe CHD groups.

Clinical Characteristics	Mild CHD Group (N = 593,048)	Severe CHD Group (N = 296,524)	ASD
Sex			0.0000
Male	385,810 (65.05%)	192,905 (65.05%)	
Female	207,238 (34.95%)	103,619 (34.95%)	
Age			0.0009
<40	15,667 (2.64%)	7693 (2.60%)	
40–49	47,793 (8.04%)	24,844 (8.39%)	
50–59	115,373 (19.45%)	57,283 (19.32%)	
60–69	178,836 (30.17%)	88,296 (29.77%)	
>=70	235,379 (39.69%)	118,408 (39.93%)	
Occupation			0.0012
Government employee	28,914 (4.90%)	12,882 (4.32%)	
Worker	298,005 (50.23%)	148,761 (50.20%)	
Farmer and fisherman	121,002 (20.45%)	62,890 (21.22%)	
Low-income	7800 (1.28%)	3557 (1.21%)	
Others	137,397 (23.14%)	68,434 (23.06%)	
Co-morbidity			
Hypertension	387,379 (65.32%)	228,264 (76.38%)	0.0197
DM	179,575 (30.28%)	129,344 (43.62%)	0.0362
Hyperlipidemia	252,164 (42.52%)	172,251 (58.09%)	0.0276
Cerebrovascular disease	55,035 (9.28%)	38,044 (12.83%)	0.0155
Peripheral vascular disease	16,012 (2.70%)	10,912 (3.68%)	0.0098
Rheumatoid arthritis	6998 (1.18%)	3884 (1.31%)	0.0004
Sjogren syndrome	8065 (1.36%)	4389 (1.44%)	0.0004
Co-medication			
Non-steroid anti-inflammatory drug	353,872 (59.67%)	178,033 (60.04%)	0.0003
Systemic corticosteroids	106,867 (18.02%)	64,494 (21.75%)	0.0034
Biguanides	112,679 (19.00%)	72,293 (24.38%)	0.0062
Dipeptidyl peptidase-4 inhibitor	64,939 (10.95%)	50,202 (16.93%)	0.0267
Glucagon-like peptide-1 agonists	949 (0.16%)	1127 (0.38%)	0.0010
Sodium–glucose cotransporter-2 inhibitors	8718 (1.47%)	6909 (2.33%)	0.0009
Statin	225,002 (37.94%)	140,226 (47.29%)	0.0388

ASD: absolute standard difference; CHD: coronary heart disease; DM: diabetes mellitus; N: number.

**Table 2:** The risk of blepharitis between the two groups.

Event	Mild CHD Group	Severe CHD Group	*p* Value
Blepharitis			
Person–months	20,253,262	9,716,179	
Event	22,161	15,369	
Crude HR (95% CI)	Reference	1.274 (1.051–1.885)	
aHR (95% CI)	Reference	1.275 (1.051–1.912) *	0.0285 *
Severe blepharitis			
Person–months	20,570,555	9,861,096	
Event	9597	4500	
Crude HR (95% CI)	Reference	1.077 (0.943–1.213)	
aHR (95% CI)	Reference	0.981 (0.945–1.020)	0.3453

aHR: adjusted hazard ratio; CHD: coronary heart disease; CI: confidence interval. * denotes a significant difference between the two groups.

**Table 3:** Subgroup analysis stratified by sex.

Event	aHR	95% CI	*p* for Interaction
Blepharitis			0.4571
Male	1.160	0.960–1.592	
Female	1.303	1.064–2.043	
Severe blepharitis			0.2671
Male	0.999	0.952–1.048	
Female	0.958	0.901–1.019	

aHR: adjusted hazard ratio, CI: confidence interval.

**Table 4:** Subgroup analysis stratified by age.

Event	aHR	95% CI	*p* for Interaction
Blepharitis			0.0115 *
<60	0.917	0.872–1.265	
60–69	1.046	0.905–1.788	
>70	1.432	1.201–2.171	
Severe blepharitis			0.5498
<60	0.956	0.886–1.030	
60–69	0.958	0.896–1.025	
>70	1.016	0.959–1.076	

aHR: adjusted hazard ratio, CI: confidence interval. * denotes a significant difference between the two groups

## Data Availability

The data applied in the present study are not available due to the policy of the National Health Insurance Bureau of Taiwan.
